# Mitochondrial DNA analysis of 114 hairs measuring less than 1 cm from a 19-year-old homicide

**DOI:** 10.1186/2041-2223-3-12

**Published:** 2012-07-13

**Authors:** Terry Melton, Gloria Dimick, Bonnie Higgins, Michele Yon, Charity Holland

**Affiliations:** 1 Mitotyping Technologies, 2565 Park Center Boulevard, Suite 200, State College, PA, USA

**Keywords:** Casework, mtDNA, Hair, Analysis, Small, Aged, Degraded

## Abstract

**Background:**

Mitochondrial DNA analysis is typically applied to degraded skeletal remains and telogen or rootless hairs. Data on the application of the method to very small hairs less than 0.5 cm from an age-matched and -challenged sample set are lacking.

**Methods:**

One hundred fourteen hairs sized less than 1 cm from a 1993 case were analyzed for mitochondrial DNA according to laboratory standard operating procedures. For some hairs, a screening approach was applied, which permitted some samples, such as victim hairs on victim clothing, to be eliminated from the process quickly. Degraded samples were amplified with “mini-primers,” and 12S species testing was applied when non-human hairs were encountered.

**Results:**

Partial to full control region human mitochondrial DNA profiles or species identifications (non-human species) were obtained from 93% of hairs under 1 cm, 92% of hairs under 5 mm, and 90% of hairs under 3.5 mm. Nineteen of 21 hairs 2 mm or less gave full or partial profiles. Among 128 hairs of all sizes tested in the case, 9 gave no results, 3 were canine in origin, and 73 did not exclude six known individuals tested in the case. Twenty-two hairs had nine additional profiles that were observed two or more times each. Twenty-one hairs showed singleton types not matching each other or any individual.

**Conclusions:**

Crime scene hairs that are both aged and small are often judged to be unsuitable for either hair microscopy or DNA analysis. This study of age-matched challenged small hairs indicates that even the smallest probative crime scene hairs are suitable for mitochondrial DNA analysis and can provide useful data.

## Background

Mitochondrial DNA (mtDNA) analysis is a routine application of DNA testing in the forensic community, supported by hundreds of peer-reviewed studies [1], SWGDAM [2] and EDNAP [3] guidelines, national and international databases (SWGDAM [4], EMPOP [5]), courtroom testimonies [6], and appellate decisions [7]. Both the public and private sectors employ laboratories to support its use in criminal and civil matters, and although standard operating procedures vary somewhat between laboratories, the general goal of all laboratories is to recover useful mtDNA profiles to test associations among individuals, crime scenes, and biological evidence.

While skeletal remains that are too degraded for STR analysis are the target of mtDNA analysis in a few cases, the last redoubt for all human hairs or hair fragments that lack a root or have a telogen root is mtDNA analysis in a skilled laboratory [8]. However, no research has ever been published on the lower size limits for successful testing of hairs, especially when those hairs represent the actual analytical challenges associated with a significant span of time since collection. We report here the results of mitochondrial DNA testing on 114 age-matched hairs that measured less than 1 cm that were collected from evidence in a homicide occurring in 1993. The significant size of this data set as well as critical control for the variable “time since collection” provides insight into the lower limits of successful mitochondrial DNA analysis in hairs. It is difficult to design a validation study for samples of this sort; therefore, reported outcomes of testing in this case will be useful to the forensic community in assessing which crime scene hairs can be forwarded for analysis with a reasonable expectation of a successful outcome.

## Methods

All identifying information about samples described here from a 1993 homicide has been removed, and permission to report and summarize the case results was solicited from the client prior to preparation of this manuscript. Hairs of all sizes were collected by the submitting agency from clothing and other crime scene items beginning in approximately 2008; none of these items had been examined since the date of the crime. The submitting agency performed a comprehensive microscopic analysis on all hairs, keeping those with anagen or catagen roots for nuclear DNA analysis in their local facility and submitting all others to Mitotyping Technologies for mtDNA analysis. The hairs were analyzed in 12 separate submissions that occurred between September 2008 and May 2010. Seven reports were provided by Mitotyping to the submitting agency, with the final report issued in June 2010. Known samples of hairs or buccal swabs from eight individuals were submitted for comparison, and known mtDNA profiles were developed early in the testing on separate dates from any of the profiles of the questioned samples. Known samples were collected according to standard collection guidelines of the national law enforcement agency that submitted the case for analysis.

Laboratory methods for mtDNA analysis of the hairs have been previously reported [8]. In addition to otherwise routine handling, these very small hairs presented challenges for manipulation that necessitated special lighting, magnification, and extra-fine-tipped forceps. Once cleaning and extraction was begun, the hair was visualized constantly throughout the process prior to grinding to avoid losing it within tubes and during transfers from multiple cleanings to grinding and incubation. Two technicians with over 10 years of experience in handling hairs were assigned to the case.

An mtDNA screening approach was implemented to save time, resources, and money for both the client and laboratory. The screening approach typically applied in large cases by this laboratory is designed to allow quick movement from profiles found in evidence samples that match known individuals or other fully profiled evidence samples to the next sample in the laboratory queue. For example, victim hairs repeatedly found on that victim’s item of clothing would not require a full profile as they would not be probative, although the first hair encountered that matched the victim would be fully profiled to confirm the full match. Similarly, the same profile repeatedly generated from a group of hairs recovered from the same location from one crime scene item, such as a hat, would not require full profiling from more than a single sample. In general, the second half of hypervariable region 1 (HV1, nucleotide positions 16160–16400) from an evidentiary sample is developed immediately after DNA extraction and then compared to the corresponding region in all the known samples, which have full profiles developed on them, or to full profiles from other questioned samples. This approach relies heavily on the relative database (SWGDAM [4] or EMPOP [5]) rarity of any given known partial profile for this region; partial profiles developed from evidence that are common or not very rare will always go on for full profiles to be developed after the initial comparison. Full profiles (HV1 15998–16400 and HV2 30–407) are developed on all new types that are observed and all types that are not matched to previously determined known sample profiles, because it is not possible *a priori* to identify which samples may become probative or exculpatory at any time during a case investigation, trial, appeal, or post-conviction investigation. For some evidentiary and known samples, where the HV1/HV2 profile is very common, additional regions (VR1 16471–16561 and VR2 424–548) [9] are developed. Full profiles are always developed for any probative samples based on the current theory of the crime.

A triage approach is also applied systematically for all hairs. After DNA extraction, one PCR amplification of HV1 16160–16400 is carried out and sequenced to assess whether this screening approach is sufficient based on the sample’s relevance to the case. For example, if a victim hair from an item of victim clothing is observed, only a this partial profile would be developed. However, a previously unobserved profile, or a profile from a probative sample located on a crime scene item (such as a hair not matching the victim on the victim’s clothing) would be developed in full. In addition, this first amplification gives significant information as to the quality and quantity of the extraction product. If this amplification does not produce a product, two mini-primer pairs encompassing the second half of HV1 are used for amplification to determine if there is abundant but degraded mtDNA [10]. If these two amplifications fail to produce amplification product, a 12S species test is applied [11]. The 12S amplification product is sequenced, and the DNA sequence is searched in GenBank to determine which species is represented by the hair. Each hair is analyzed individually from extraction through sequencing. All samples are accompanied by DNA extraction negative controls (reagent blanks), PCR negative controls, and an HL60 positive control throughout analysis. Contamination profiles observed in reagent blank negative controls are compared to each other, to those of laboratory staff, and those of other samples processed within the laboratory and within the case, and their occurrences are reported to the client.

## Results and discussion

In this study, 132 hairs were submitted for analysis mounted on slides with mounting medium securing the hairs to the slides. Each slide was labeled with client information, and each hair was given a unique laboratory identifier after arrival. Four hairs were not tested because of their fine diameter and difficulty visualizing them while still mounted on the slide. Of the remaining 128 hairs, portions of 1.5 - 2 cm (cm) were taken from four hairs, ten more hairs of size 1 cm to 2.2 cm were consumed, and an additional 114 hairs < 1 cm in size were consumed in testing. One hundred one (101) of the 132 hairs were 0.5 mm to 5 mm in size. All hairs tested by the laboratory had either naturally shed roots or no roots. Table 1 shows the distribution of samples and the results for each category.

**Table 1:** Outcomes (count and frequency) of mitochondrial DNA analysis in 114 small casework hairs from 1993

Size	No results	Screened	Full profiles	Partial profile	Mini-primer sets used	12S (canine)	Total
≥ 5 mm to <1 cm	0	6	6	0	1	0	13 (0.11)
< 1 mm to 5 mm	8	40	34	8	8	3	101 (0.88)
Total N (freq.)	8 (0.07)	46 (0.40)	40 (0.35)	8 (0.07)	9 (0.08)	3 (0.03)	114

Hairs in the “no results” category underwent amplification for part of HV1, two amplifications with mini-primers, and 12S species analysis, and yielded no amplification or sequence product. Hairs in the “screened” category had only the last half of HV1 amplified and sequenced; these samples in theory could have had full profiles developed if necessary. Partial profiles were developed using two or more regular primer pairs (not mini-primers), and had either all of HV1 developed or HV1 and part of HV2 obtained. Samples designated in the “mini-primer” category required mini-primers to develop a partial profile. Three hairs were consistent with *Canis familiaris* as a result of 12S analysis.

**Figure 1:**
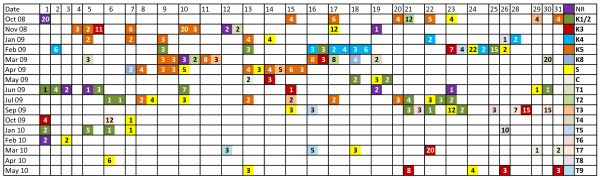
**Distribution of failures to exclude 128 individual hairs over the dates of testing based on date of DNA extraction of each hair.** Day of the month on the top line; on some days more than one sample was extracted. *Dark colors* are failures to exclude known individuals (K1-K5, K8). *Lighter colors* are types occurring more than one time (T1-T9) that do not match known individuals. Number within the box is size of hair in millimeters rounded up to the closest millimeter. *C* = canine, *S* = singleton types, *NR* = no results. *Color key* on the *right.*

Eight known samples, including elimination samples from two staff members from the submitting laboratory, were analyzed early in the hair submission process to allow for screening of evidence samples against the known profiles. Profiles that failed to exclude case-related known individuals were obtained from the questioned hairs during the full range of the testing period at random intervals interspersed with profiles not observed in any of the known individuals. During the course of testing, numerous other casework samples were handled in the laboratory and the profiles of the hairs in this case were not observed in questioned or known samples from other cases being handled contemporaneously. Figure 1 shows the distributions of failures to exclude interspersed with unsourced types, no results, and non-human results over the testing period.

Reagent blank (RB) contamination, defined as the presence of amplification products that could be sequenced and analyzed, was observed during case processing. All RBs were sequenced irrespective of the presence of product on the yield gel. Overall, 15 hairs had some evidence of reagent blank involvement, where 22 RB amplification products from 66 separate amplifications from these samples resulted in a sequence product. Of these, 11 PCR products contained minimal DNA from one or other of the two DNA technicians who handled the sample. All other RB types were sporadic, meaning that they were different from each other and not observed a second time. All six hairs with RBs that were not technician types had profiles different from the profile observed in the accompanying RB product. Contaminants were observed with a range of primer pairs, and one was a recurrent laboratory contaminant that had been observed in casework occasionally over the years. This indicates that it is likely due to an equipment or supply source. In one hair, the contaminant had the same profile as the sample and a repeat analysis was clean. Thirteen of 114 hairs < 1 cm experienced some degree of reagent blank contamination versus two of 14 hairs ≥ 1 cm in size, a statistically insignificant difference. In all cases, an interpretation was carried out as defined by the laboratory’s validation studies on contamination. No profiles from the case or from other cases handled contemporaneously were observed in the reagent blanks.

A validation experiment for simultaneously testing “old” and “small” hairs is difficult to design and implement. For example, it would be difficult to assemble a sample set such as the hairs tested in this case, with 19-year-old hairs from numerous individuals that had been stored for many years. Figure 1 allows an analysis of sample profile authenticity. Over the 20-month testing period, known individuals’ profiles were observed interspersed with other unassociated types (to date) that occurred either singly or multiple times. For some samples the profile of a known individual was observed prior to the testing of the known sample, for example, K8 was tested late in the case, whereas two hairs matching K8 were tested early. Clusters of types generally indicated that a group of hairs was collected from the same item of clothing; however, most known types are scattered throughout the length of the analysis. Singleton unmatched types (yellow) are evenly distributed throughout the testing, as are samples giving no results (purple). The first hair tested, 2 cm in size, failed to give a result, whereas the remaining eight hairs giving no result were all 2 mm or less in size. However, the frequency of “no result” hairs was not different between the two groups, though sample sizes were small (0.07 for hairs > 1 cm versus 0.07 for hairs ≤ 1 cm). Samples extracted on adjacent days in the same extraction space often gave disparate types, arguing against cross- or carryover-contamination, and there was no correlation with the appearance of known types in the evidence with timing of the analysis of the known samples. Neither technician types nor types from other cases being handled in the same time frame were observed in the questioned samples. Based on a history of the case, the presence of many types from clothing evidence was not unexpected.

## Conclusions

In 2005, this laboratory reported that 80% of casework hairs ≤ 1 cm gave a full or partial profile ([8]; *N* also = 114); however, the sample set reported here is age-matched for one particular case from 1993, providing an important control of the variable “time since collection,” while the sample set reported then contained hairs of all ages. For this sample set, partial to full control region human mtDNA profiles or species identifications (non-human species) were obtained from 93% of hairs < 1 cm, 92% of hairs < 0.5 cm, and 90% of hairs < 3.5 mm. Nineteen of 21 hairs ≤ 2 mm gave full or partial profiles. In the 2005 study, where most of the 114 hairs were greater in size than 0.5 cm, it was reported that 10 out of 12 hairs ≤ 0.5 cm gave a partial or full profile; these ranged in age from about one to 22 years since time of collection. In the present study, this much larger data set of the smallest hairs (*N* = 101 hairs < 0.5 cm) indicates that 92% of age-matched hairs < 0.5 cm from 1993 can provide either partial or full mitochondrial DNA profiles.

The information that mtDNA profiles can be obtained from very small hairs nearly 2 decades old will be useful to crime scene and trace evidence personnel. With little published data on very small hairs, our clients often assume that a 2-cm hair is always necessary or desirable for mitochondrial DNA analysis. Some laboratories do not accept hairs less than 2–3 cm. In our experience, longer hairs with a history of environmental degradation may not give results; therefore, size is the least important consideration in testing crime scene hairs. In this case, comparison microscopy and STR analysis were not possible, and this large sample set provided a more realistic opportunity to judge the challenges to testing and opportunities for outcomes than any designed study could provide. Equally small hairs less than 2 decades old should work as well or better for mtDNA analysis than those described here, and it is possible if not likely that many hairs older than 2 decades can be analyzed with some success as well.

The ability to apply DNA profiling to extremely small hairs introduces new questions for the forensic community, because it expands the universe of samples that can be collected from a crime scene. Since this laboratory’s inception, we have counseled that submitted samples must have relevance to any and all theories of the crime, based on two features of mtDNA: its non-uniqueness (and limited utility in court) and the innocent nature of most hair deposition, as opposed to the deposition of samples such as blood and semen. In many cases, new theories of a specific crime develop over time, and it is not possible to know *a priori* which one will take center stage in the prosecution of a case. As sample sizes get smaller, and the triers of fact, attorneys, and law enforcement develop understanding of the power of DNA testing, it is incumbent on the forensic community to select, submit, screen, and profile the most valuable and relevant samples, while educating the public about DNA’s limitations.

## Competing interests

The authors comprise professional staff of a commercial forensic DNA testing facility, Mitotyping Technologies, which is financing the production of this manuscript. The lead author, TM, is a part owner of Mitotyping Technologies.

## Authors’ contributions

TM and CH were forensic examiners for the case, edited the data, and supervised the technical staff. GD, BH, and MY carried out the laboratory mitochondrial DNA analyses. TM and CH compiled and analyzed the data. TM drafted the manuscript. All authors read and approved the final manuscript.

## References

[B1] Ruiz-Pesini E Lott MT Procaccio V Poole JC Brandon MC Mishmar D Yi C Kreuziger J Baldi P Wallace DC An enhanced MITOMAP with a global mtDNA mutational phylogeny Nuc Acids Res 2007 35 D823 D828 10.1093/nar/gkl927 PMC1781213 17178747

[B2] Scientific Working Group on DNA Analysis Methods Guidelines for mitochondrial DNA (mtDNA) nucleotide sequence interpretation For Sci Comm 2003 5

[B3] Tully G Bär W Brinkmann B Carracedo A Gill P Morling N Parson W Schneider P Considerations by the European DNA profiling (EDNAP) group on the working practices, nomenclature, and interpretation of mitochondrial DNA profiles For Sci Int 2001 124 83 91 10.1016/s0379-0738(01)00573-4 11741765

[B4] Monson KL Miller KWP Wilson MR DiZinno JA Budowle B The mtDNA population database: an integrated software and database resource for forensic comparison For Sci Comm 2002 4

[B5] Parson W Brandstatter A Alonso A Brandt N Brinkmann B Carracedo A Corach D Froment O Furac I Grzybowski T The EDNAP mitochondrial DNA population database (EMPOP) collaborative exercises: organisation, results, and perspectives For Sci Int 2004 139 215 226 10.1016/j.forsciint.2003.11.008 15040920

[B6] Morrisey M Mitochondrial DNA (mtDNA) Cases http://www.denverda.org/DNA/Mitochondrial_DNA_Legal_Decisions.htm

[B7] United States Court of Appeals Sixth Circuit: United States v. Beverly http://caselaw.findlaw.com/us-6th-circuit/1122482.html

[B8] Melton T Nelson K Forensic mitochondrial DNA analysis of 691 casework hairs J For Sci 2005 50 73 80 15830999

[B9] Lutz S Wittig H Weisser H-J Heizmann J Junge A Dimo-Simonin N Parson W Edelmann J Anslinger K Jung S Is it possible to differentiate mtDNA by means of HVIII in samples that cannot be distinguished by sequencing the HVI and HVII regions? For Sci Int 2000 113 97 101 10.1016/s0379-0738(00)00222-x 10978608

[B10] Gabriel MN Huffine EF Ryan JH Holland MM Parsons TJ Improved mtDNA sequence analysis of forensic remains using a "mini-primer set" amplification strategy J For Sci 2001 46 247 253 11305426

[B11] Melton T Holland C Routine forensic use of the mitochondrial 12 S ribosomal RNA gene for species identification J For Sci 2007 52 1305 7 10.1111/j.1556-4029.2007.00553.x 17868265

